# The spatial overlap between left atrial epicardial adipose tissue and fibrosis is not associated to clinical stage of atrial fibrillation

**DOI:** 10.1038/s41598-024-75428-8

**Published:** 2024-10-22

**Authors:** Iulia Skoda, Markus Henningsson, Lars O. Karlsson, Carl-Johan Carlhäll

**Affiliations:** 1 Department of Cardiology in Linköping, and Department of Health, Medicine and Caring Sciences, Linköping University, 58183 Linköping, Sweden; 2 Unit of Cardiovascular Sciences, Department of Health, Medicine and Caring Sciences, Linköping University, Linköping, Sweden; 3 Center for Medical Image Science and Visualization (CMIV), Linköping University, Linköping, Sweden; 4 Department of Clinical Physiology in Linköping, and Department of Health, Medicine and Caring Sciences, Linköping University, Linköping, Sweden

**Keywords:** Atrial fibrillation, Epicardial adipose tissue, Atrial fibrosis, Left atrium, MRI, Clinical trials, Interventional cardiology

## Abstract

Left atrial (LA) epicardial adipose tissue (EAT) and wall fibrosis are both proven to contribute to the pathogenesis and progression of atrial fibrillation (AF). The theory of LA wall fibrosis induction by local EAT infiltration, paracrine secretions, and activation of the inflammatory process is strongly advocated, but the imaging evidence for anatomical proximity of the two tissue types and its association to AF stage is lacking. Accordingly, the aim of the study was to analyse the spatial overlap between LA EAT and adjacent wall fibrosis using 3D Dixon water-fat separated late gadolinium enhancement (LGE-Dixon) MRI and correlate the findings with the clinical AF stage. Forty-two AF patients (18 paroxysmal, 10 persistent, and 14 permanent) and nine non-AF patients were scanned. The permanent AF patients had greater LA volume and EAT than the paroxysmal group. The LA fibrosis area showed the same trend. The LA EAT-fibrosis overlap area was small and there was no significant difference between the three AF stages. There was no significant relationship between LA EAT- fibrosis overlap area and AF type. The findings shed light on the complex interplay between LA fibrosis and EAT during the progression from paroxysmal to permanent AF.

## Introduction

Atrial fibrillation (AF) is the most common arrhythmia and poses a significant burden to patients and healthcare systems globally^1^. AF generates unpleasant symptoms, and a high arrhythmia burden is associated with higher risks of stroke, mortality, and hospitalization for heart failure worldwide. All these aspects are amplified by the aging population and the prospect of an increasing AF burden in the coming decades, disproportionally affecting the elderly^2^. The pathophysiological process of AF can be complex and may include, for instance, the interaction of epicardial adipose tissue (EAT), inflammation and tissue changes of the left atrial (LA) wall leading to fibrosis^3,4^. The old concept of a vicious circle of electrophysiologic and structural changes and the dogma of “AF begets AF”^5^ are strongly supported by more recent studies^6^, but the starting point of the process is not clearly defined. The disturbed blood flow pattern during the initial short AF episodes may induce atrial endothelial microinjury, leading to the migration of macrophages into atrial tissues, triggering an inflammation loop^7^. On the other hand, as a result of epicardial adipocytes accumulation (forming EAT) and adipokine secretion, immune cells are recruited and activate fibroblasts. Ultimately, fibro-fatty infiltration promotes AF progression^8,9^. Recently, Abe et al. showed^9^ that the histological vicinity of EAT and fibrosis is greater in the left atrial wall of AF patients compared to non-AF patients. However, with the advancement of new imaging techniques for analysis of EAT and LA fibrosis, their spatial distribution and overlap can now be obtained non-invasively, which could allow a deeper understanding of the pathophysiology of the AF disease in-vivo.

Late gadolinium enhancement (LGE) cardiac magnetic resonance imaging (MRI) technique is the current noninvasive reference standard for the assessment of myocardial fibrosis^10^. The clinical applicability has widened through specific sequences, expanding to pre-procedural assessment of arrhythmogenic substrate^11,12^, visualization of radiofrequency-induced ablation lesions^13^, and the detection of epicardial fat^10,14^. However, even though Dixon water-fat separated late gadolinium enhancement (LGE-Dixon) cardiac MRI has previously been used to simultaneously visualize EAT and LA fibrosis^15^, quantitative data on the anatomical overlap is scarce.

The aim of the present study was to analyse the spatial overlap between EAT and adjacent LA wall fibrosis, using 3D Dixon water-fat separated LGE MRI and correlate the findings with the clinical AF stage.

## Results

**Fig. 1:**
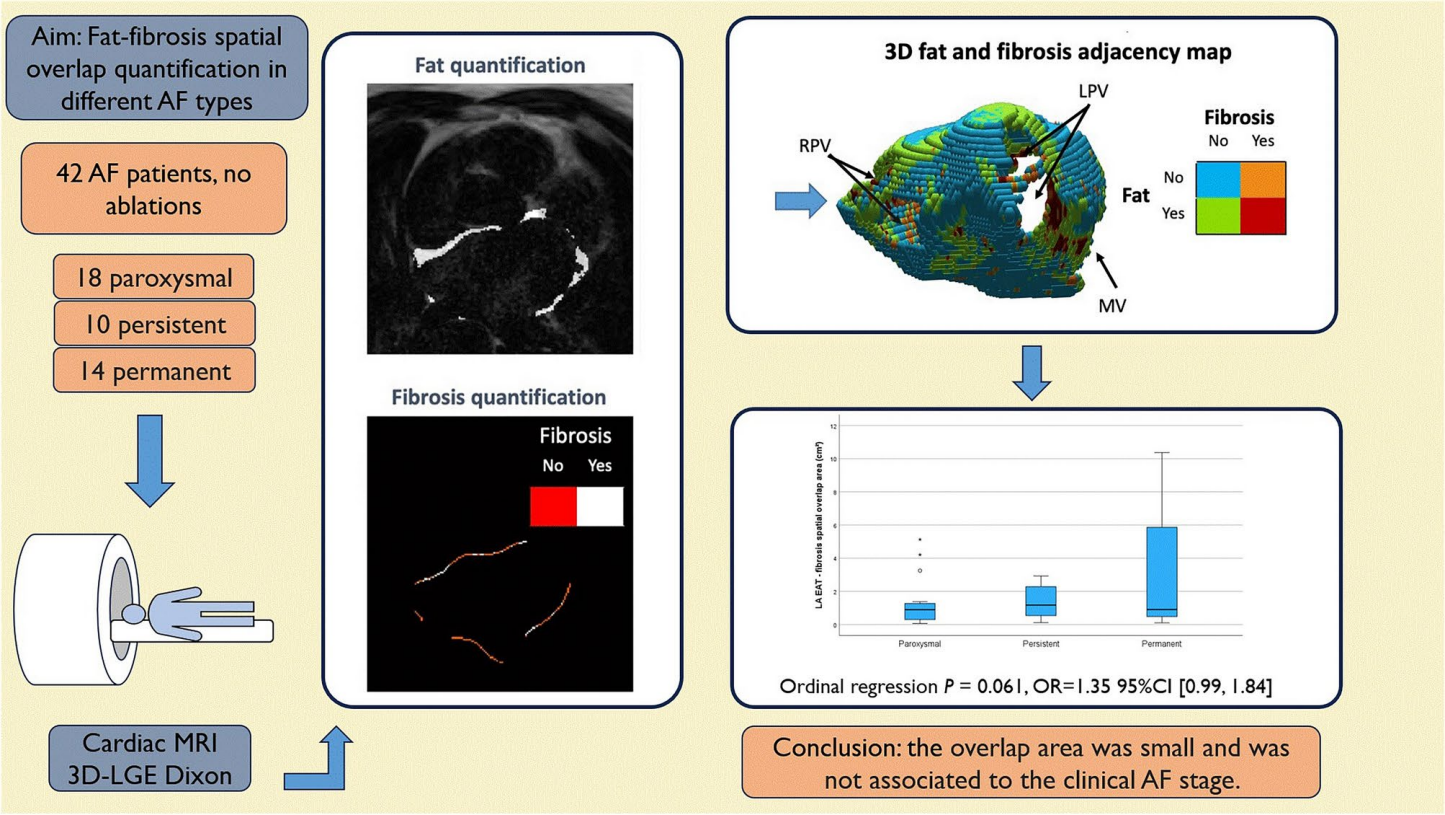
Graphical abstract illustrating an overview of the aim, methods, results, and conclusion. The 3D map was created with MATLAB R2021b (The MathWorks, Natick, Massachusetts, USA, https://uk.mathworks.com/products/matlab.html).

A summary of the used method and an overview of the results are presented in Fig. 1.

The clinical characteristics of included patients are summarized in Table 1, and their cardiovascular background is reported in Table 2. In total, 42 AF patients (age 65.8 ± 9 years) and 9 non-AF patients (age 61.3 ± 9.4 years) underwent MRI scanning. Thirty-two patients had sinus rhythm (SR) during the scan, while the other 19 had AF. There were no differences in the baseline characteristics between the four groups except for left ventricular (LV) ejection fraction (EF), which was highest in patients with paroxysmal AF and LV end-diastolic volume index (LVEDVI), which was largest in the non-AF group.

**Table 1:** Baseline characteristics of the population.

Atrial fibrillation type	Non-AF	Paroxysmal	Persistent	Permanent	*P*
N	9	18	10	14	
Age, y	61.3 ± 9.4	62.9 ± 11.4	66.8 ± 6.1	68.7 ± 6.1	0.167
Male sex, n (%)	5(56)	10(56)	7(70)	11(79)	0.514
BMI, kg/m^2^	25.4 ± 4.1	27.3 ± 3.5	27,1 ± 1.8	27.6 ± 3.6	0.462
BSA, m^2^	1.93 ± 0.2	2.03 ± 0.3	2.07 ± 0.2	2.05 ± 0.2	0.501
Heart rate, beats/min	68.1 ± 1	60,3 ± 20	64 ± 19	73 ± 14	0.209
SR during the MRI scan, n	9	16*	7	0	** < 0.001**
LVEDVI, mL/m^2^	105 ± 38	71 ± 15	85 ± 14	80 ± 20	**0.004**
LVEF, %	52 ± 17	60 ± 7*	52 ± 10	48 ± 12	**0.023**

BMI: body mass index; BSA: body surface area; LVEF: left ventricular ejection fraction.

LVEDVI: left ventricular end-diastolic volume index; SR: sinus rhythm;

*P < 0.02 versus permanent group.

**Table 2:** Cardiovascular background of the population.

	Non-AF	Paroxysmal AF	Persistent AF	Permanent AF	Total	P*
N	9	18	10	14	51	
Hypertension	1	10	5	10	26	**0.042**
Heart failure	4	2	5	9	20	**0.017**
Hypertrophic cardiomyopathy	1	0	0	0	1	0.190
Ischemic heart disease	1	1	3	5	10	0.130
Previous stroke	0	2	1	3	6	0.476
Diabetes mellitus	0	3	0	2	5	0.342
Family history AF	0	3	2	0	5	0.201
Rheumatic disease	1	2	2	1	6	0.813
COPD	0	1	0	2	3	0.395
Myopericarditis	1	2	0	1	4	0.739
Thyroid disease	1	1	0	1	3	0.774
Takotsubo cardiomyopathy	1	2	0	0	3	0.418
Significant valve disease	0	0	1	1	2	0.481
Chronic kidney disease	0	0	0	1	1	0.441

*P by Pearson Chi-Square.

COPD: Chronic obstructive pulmonary disease.

MRI parameters of the LA are summarized in Table 3. LA volume and LA EAT were normally distributed, while the relative (% of the total LA wall area) and absolute fibrosis area and LA EAT- fibrosis overlap area (both relative and absolute) were not.

**Table 3:** Left atrial variables.

Atrial fibrillation type	Non-AF	Paroxysmal	Persistent	Permanent	*P*
N	9	18	10	14	
LA volume, mL	113 ± 40	124 ± 35*	159 ± 38	188 ± 54	** < 0.001**
LA volume index, mL/m^2^	59 ± 20	61 ± 15*	77 ± 20	91 ± 25	** < 0.001**
LA EAT volume, mL	13 ± 6	10 ± 6*	17 ± 12	23 ± 12	**0.004**
LA EAT volume index, mL/m^2^	6.5 ± 3	5 ± 3*	8 ± 5	11 ± 5	**0.002**
LA EAT area, cm^2^	23 ± 8	24 ± 13*	31 ± 16	38 ± 14	**0.013**
LA EAT area index, cm^2^/m^2^	12 ± 4	12 ± 6*	15 ± 7	19 ± 6	**0.012**
LA fibrosis (%) as median (iqr)	9 (8.3)	6.4 (7)	8 (5)	7 (10)	0.182
LA fibrosis (%) as median (iqr), IIR > 1.2	11.6 (16.3)	8.35 (14.4)	11 (14.4)	9.6 (10.7)	0.710
LA fibrosis (%) as median (iqr), IIR > 1.32	4.5 (8.5)	2.04 (5.5)	2.2 (7.9)	2.2 (5.4)	0.957
LA fibrosis area (cm^2^) as median (iqr)	7.7 (9.6)	5.6 (6)	9.5 (6)	7.9 (15)	0.137
LA EAT—fibrosis overlap (%) as median (iqr)	1.2 (2.4)	0.8 (1.3)	1.1 (1.4)	0.7 (4)	0.685
LA EAT—fibrosis overlap (%) as median (iqr), IIR > 1.2	1.03 (4.2)	1.24 (3.2)	0.89 (3.4)	1.8 (3.5)	0.897
LA EAT—fibrosis overlap (%) as median (iqr), IIR > 1.32	0.21 (2.2)	0.16 (1)	0.09 (1.6)	0.32 (1.2)	0.471
LA EAT—fibrosis overlap area (cm^2^) as median (iqr)	1.3 (1.9)	0.9 (1)	1.2 (2)	0.9 (5.6)	0.269

EAT: epicardial adipose tissue; LA: left atrial.

*P < 0.02 versus permanent group.

**Fig. 2:**
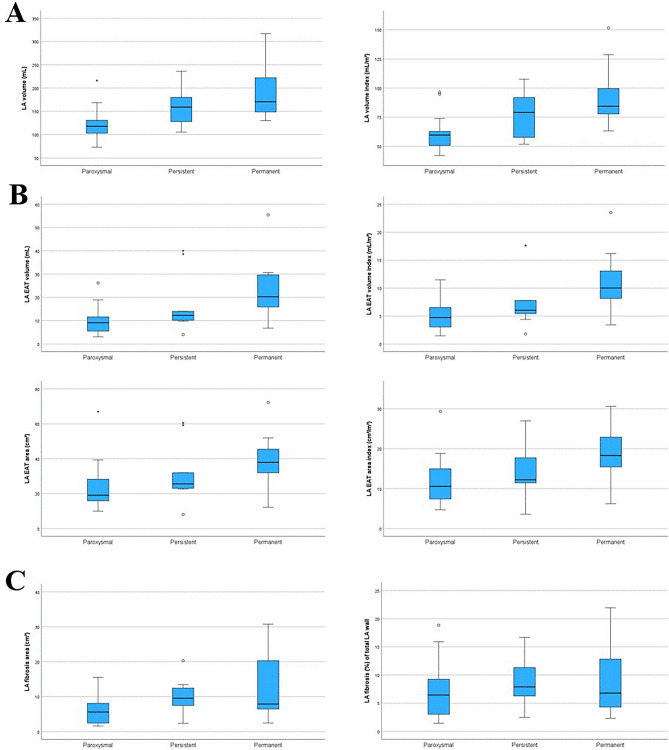
Distribution of left atrial (LA) volume (**A**), epicardial adipose tissue (EAT) (**B**), and LA wall fibrosis (**C**), across the three atrial fibrillation (AF) types, represented as median and interquartile range. Extreme outliers are marked with an asterisk (*), mild outliers are marked with a circle (O).

The absolute and BSA indexed values of the LA volume, LA EAT volume, and LA EAT area, increased from paroxysmal to permanent AF (Fig. 2).

The absolute LA fibrosis areas reported as median (interquartile range) were 5.6 (6) cm^2^, 9.5 (6) cm^2^ and 7.9 (15) cm^2^ for paroxysmal, persistent, and permanent AF, respectively (*P* = 0.137). The LA fibrosis relative areas calculated as percent of the total LA wall area, were 6.4 (7) %, 8 (5) % and 7 (10) % for paroxysmal, persistent, and permanent AF, respectively (*P* = 0.182) (Fig. 2).

**Fig. 3:**
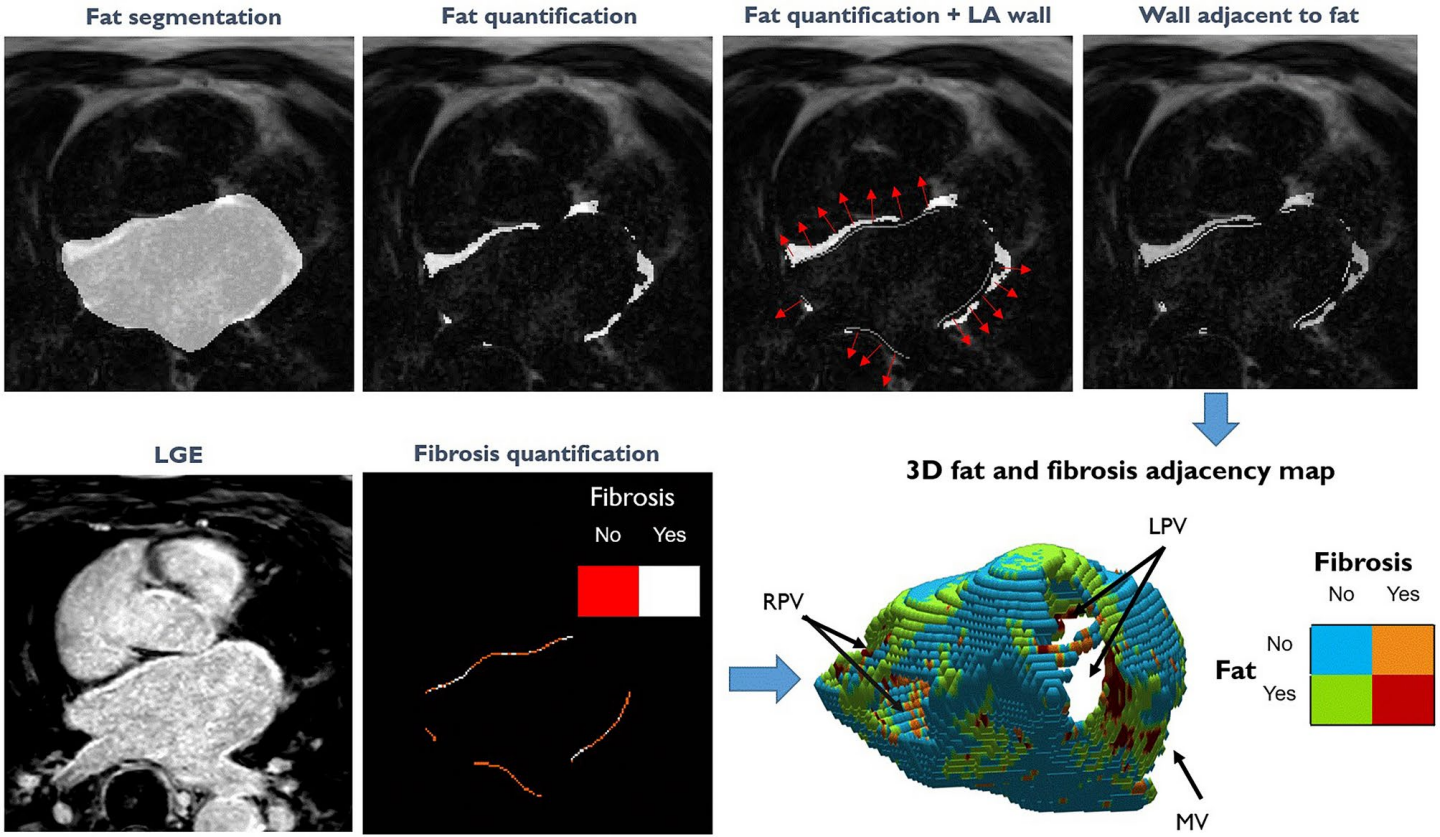
An example of the current method and a generated 3D left atrial map. LA = left atrial; LGE = late gadolinium enhancement; LPV = left pulmonary veins; RPV = right pulmonary veins; MV = mitral valve; EAT = epicardial adipose tissue; Fat = LA EAT. The 3D map was created with MATLAB R2021b (The MathWorks, Natick, Massachusetts, USA, https://uk.mathworks.com/products/matlab.html).

**Fig. 4:**
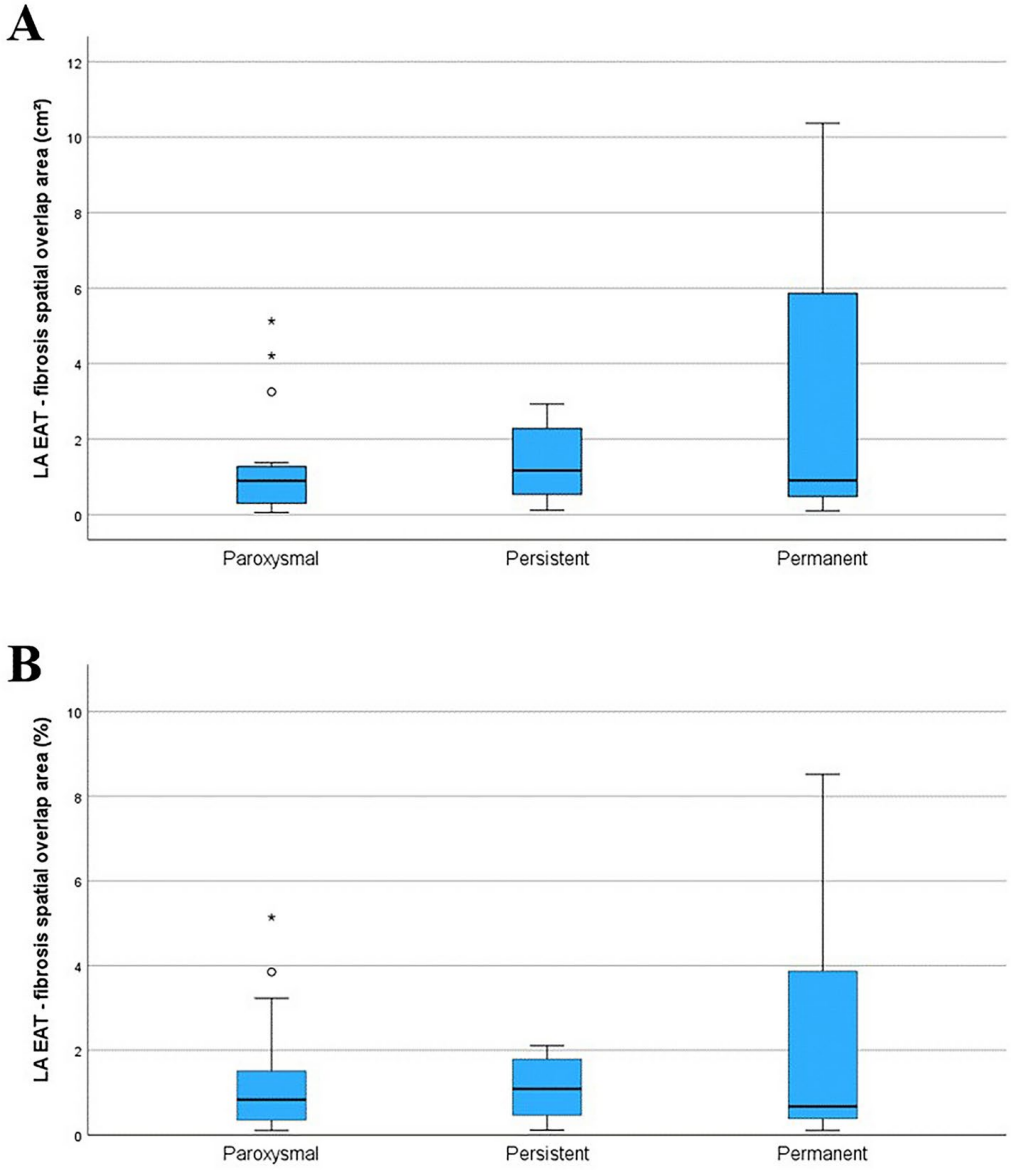
Distribution of left atrial (LA) epicardial adipose tissue (EAT)—fibrosis spatial overlap area, across the three atrial fibrillation (AF) types, represented as median and interquartile range of the absolute area (**A**) and relative area (**B**). Extreme outliers are marked with an asterisk (*), mild outliers are marked with a circle (O).

An example of the used LA EAT—fibrosis overlap method and a 3D representation of the resulted LA wall map is depicted in Fig. 3. The LA EAT—fibrosis overlap areas, both as percentage of the total LA wall area and as absolute area, were small compared to the total LA fibrosis areas (*p* < 0.001 Wilcoxon signed rank test). The median (interquartile range) value comparison of these absolute overlapping areas for the different AF types [0.9 (1) cm^2^, 1.2 (2) cm^2^ and 0.9 (5.6) cm^2^ for paroxysmal, persistent, and permanent AF, respectively] showed no significant difference (*P* = 0.269). The comparison of the relative LA EAT—fibrosis overlap areas for the different AF types [0.8 (1.3)%, 1.1 (1.4)% and 0.7 (4)% for paroxysmal, persistent, and permanent AF, respectively] showed no significant difference (*P* = 0.685) either (Fig. 4).

The ordinal logistic regression analysis performed on the three AF groups revealed that both indexed LA volume and indexed LA EAT volume were able to predict AF type (*P* < 0.001, OR = 1.061 95%CI [1.03, 1.1] and *P* = 0.003, OR = 1.28 95%CI [1.09, 1.5], respectively). The analysis was similar regarding the absolute LA fibrosis area (*P* = 0.021, OR = 1.13 95%CI [1.02, 1.25]) but the prognostic relationship between LA fibrosis relative area and AF type (*P* = 0.254) or between LA EAT- fibrosis overlap area (absolute or relative) and AF type was not significant (*P* = 0,061 and *P* = 0.227, respectively). The ordinal logistic analysis between AF type and relative fibrosis and relative LA EAT-fibrosis overlap areas quantified according to the IIR method, resulted in *P* > 0.695, for both IIR > 1.2 and IIR > 1.32.

The logistic regression analysis of LA fibrosis and LA EAT-fibrosis overlap area, dividing all AF patients based on the median LVEF value (57%) showed no significant association, see Table 4.

**Table 4:** Logistic regression for left atrial (LA) fibrosis area and LA epicardial adipose tissue (EAT)-fibrosis overlap area, left ventricular ejection fraction (LVEF) value above/below 57%

Logistic regression *P*, odds ratio, confidence interval	Variable
(*P* = 0.632, OR = 1.028 95%CI [0.917, 1.153])	LA fibrosis area, valve signal intensity
(*P* = 0.855, OR = 1.031 95%CI [0.740, 1.438])	LA EAT-fibrosis overlap area (%),valve signal intensity
(*P* = 0.905, OR = 0.997 95%CI [0.948, 1.048])	LA fibrosis area, IIR > 1.2
(*P* = 0.540, OR = 0.955 95%CI [0.824, 1.107])	LA EAT-fibrosis overlap area (%), IIR > 1.2
(*P* = 0.947, OR = 0.997 95%CI [0.972, 1.073])	LA fibrosis area, IIR > 1.32
(*P* = 0.549, OR = 0.930 95%CI [0.732, 1.180])	LA EAT-fibrosis overlap area (%),IIR > 1.32

## Discussion

This study presents a comprehensive evaluation of the LA EAT—wall fibrosis co-localization in different types of AF. The main finding was the relatively small magnitude of the LA EAT-fibrosis spatial overlap area (both relative and absolute values) and the lack of its correlation with the clinical AF stage with respect to paroxysmal, persistent and permanent AF, irrespective of the fibrosis quantification method.

This finding is somewhat surprising, as the previous published literature shows a positive correlation between AF progression and both LA EAT and wall fibrosis. Our findings confirm the LA EAT volume gradual increasing pattern with the progression of AF. Regarding the LA fibrosis area, the values follow the same trend, but do not reach the significance level. This might be explained by the variability in the extent of fibrosis in patients with paroxysmal and persistent AF, with some of the paroxysmal AF patients having substantial fibrosis as well as some of the persistent AF patients showing mild fibrosis^11,16–19^. Furthermore, the assessment of LA wall fibrosis can be more challenging than assessment of LA EAT from a methodological point of view^20^. The low values of fibrosis quantified according to the IIR method and using a threshold of 1.32 might relate to the fact that none of the patients was ablated before the MRI scan, thus no suspicion of dense scar^21^.

The molecular interaction between LA EAT and fibrosis, and the notion that LA EAT initiates the fibrotic remodeling through inflammation^4,6,9^, suggests their adjacent coexistence during AF disease progression. One can speculate on the positive correlation of LA EAT and LA wall fibrosis volumes in the late AF stages, but the inflammation process might also “consume” the EAT volume by inducing high catabolism, since the rates of lipolysis and insulin-induced lipogenesis are higher in epicardial fat than in other visceral fat depots^22^. The process may lead to low EAT quantities in individuals with advanced atrial disease, marked by high levels of LA wall fibrosis. This perspective highlights the importance of LA EAT—fibrosis interplay analysis in AF patients at different stages of disease. In this study, the trends of the correlation analysis suggested increased LA EAT volume and LA fibrosis area with AF progression. However, the overlapping LA EAT—fibrosis areas, which might be seen as the histological ground for local interaction, were small, even in patients with high wall fibrosis and LA EAT areas. The anatomical proximity of LA EAT and LA fibrosis in AF patients was analysed by Chahine et al. with a slightly different method (for every point on the LA surface, the authors measured the minimal distance to the nearest point within the EAT volume) concluding that LA EAT regions do not colocalize with LA fibrotic areas, favoring a systemic or endocrine mechanism rather than the local EAT infiltration^23^. The study was performed on a cohort with similar baseline characteristics, yet larger, however, the analysis did not focus on the three specific clinical AF types. The correlation between LA EAT index and LA fibrosis improved slightly in the overweight and obese participants indicating that the LA EAT may have a greater effect on fibrosis in these subsets of patients. The latter analysis is not relevant for our cohort, since we have few obese participants.

In another study, Mahajan et al., address the LA EAT-LA wall fibrosis overlap in an indirect way (electroanatomic mapping with previously standardized definitions of scar by regions of low voltage, reduced conduction velocity and/or electrogram fractionation)^24^. The authors analysed a cohort of 26 slightly younger AF patients (18 paroxysmal and 8 persistent) and divided the cohort according to BMI (under or above 27 kg/m^2^). The electroanatomic mapping was performed during sinus rhythm. They defined specific LA wall regions for the LA EAT distribution and their conclusion was that the EAT measures correlated better with LA conduction abnormalities, compared to BMI. In particular, LA EAT was the best predictor of conduction abnormalities in the contiguous posterior LA wall, a region in close spatial relationship with the fat pad, and raise the possibility of fat infiltration of the LA myocardium by contiguous fat depots, but different input from the autonomic ganglia that reside in the fat pads could not be excluded.

Zghaib et al.^25^, investigated the association of LA EAT (computed tomography-based analysis) with adjacent myocardial substrate. The LA wall fibrosis was again defined indirectly by electroanatomic mapping in sinus rhythm. The authors analysed a cohort of 30 slightly younger AF patients (22 paroxysmal and 8 persistent/long standing persistent) and reported a positive association between electrogram fractionation in sinus rhythm and age, male gender, diabetes, hypertension, and LA EAT. BMI and age were associated with the presence of LA EAT. However, LA EAT was not a statistical mediator of the association of clinical variables with atrial scar.

None of the studies mentioned above report a specific quantification of the LA EAT—fibrosis spatial overlap area, nor do they focus on the specific AF clinical stages or AF progression.

Based on the histological and electrophysiological evidence of LA EAT and LA fibrosis interplay in the debut and progression of AF, there have been attempts to address these specific areas during AF ablation. The fat oriented ablation focused on high-dominant frequency sites covered with LA EAT^26,27^, while the fibrosis oriented approach targeted electrograms showing complex fractionated activity^28^ or MRI-based detection of LA fibrosis^29^. Some of these attempts were promising^30^, while others gave a more pessimistic perspective^20,28^. Long term freedom of AF after ablation is still a clinical relevant challenge, emphasizing the necessity of further research and a deeper understanding of AF pathophysiology and the process of LA EAT- fibrosis interaction in AF progression.

Regarding the findings in the non-AF group, they emphasize that LA fibrosis is not specific to AF, but part of the atrial myopathy complex. While the LA volume, LA EAT volume and LA EAT area, progress from non-AF to permanent AF, LA fibrosis and LA EAT-fibrosis overlap seem to reach the same levels in the non-AF group as in persistent and permanent AF, underscoring the importance of comorbidity. However, the LA fibrosis and LA EAT-fibrosis overlap area, did not seem to relate to lower LVEF in the current population, advocating for a more sophisticated relationship between LV dysfunction and LA fibrosis.

There are some limitations in our study. A relatively small number of patients was included in this single-center study, although it is comparable to similar proof-of-concept studies utilizing advanced cardiac imaging methodology. Both segmentation of EAT and LA wall were done manually which is time consuming. AI-based techniques are emerging and seem to be promising in overcoming this problem. There are no general agreements on the fixed thresholds for EAT and fibrosis quantification in cardiac MRI, leading to some variation in results and making it challenging for centers to compare their results.

To conclude, the LA EAT—fibrosis spatial overlap area was small in patients with AF and was not associated to the clinical AF stage. The findings shed light on the complex interplay between these two important AF risk factors during the progression from paroxysmal to permanent AF and warrant further research.

## Methods

### Study participants

This prospective study was approved by the Swedish Ethical Review Authority. All participants provided written informed consent to participate. We confirm that the study was performed in accordance with relevant guidelines and regulations including the Declaration of Helsinki.

The participants were recruited between February 2019 and October 2023: either from the Department of Cardiology at Linköping University Hospital, or by having a clinical indication for a cardiac MRI at the Linköping University Hospital. Inclusion criteria: non-AF, paroxysmal, persistent, or permanent AF. Exclusion criteria: contraindication to MRI; previous catheter ablation or other cardiac surgery, more than moderate valve disease.

### Cardiac MRI protocol

All experiments were performed on a 1.5 T scanner (Philips Healthcare, Best, The Netherlands) using a 28-channel cardiac coil. A previously presented and validated LGE-Dixon sequence^15^ was performed approximately 20 min after Gadolinium-based contrast agent injection (0.2 mmol/kg gadobutrol or 0.2 mmol/kg gadoterate meglumine) with the following imaging parameters: orientation = transverse; field- of- view (FOV) = 320 × 320 × 120–140 mm^3^; spatial resolution = 1.25 × 1.25 × 2.5 mm^3^; flip angle = 20º; Repetition time(TR)/Echo time(TE)_1_/TE_2_ = 7.1/2.2/4.8 ms; pixel bandwidth = 433 Hz; acquisition window = 100 ms, linear profile order. For image acceleration, an online commercial compressed SENSE technique was used with acceleration factor 5. Water and fat images were reconstructed online using a commercially available modified Dixon (mDixon) technique. A diaphragmatic respiratory navigator with 6 mm gating window was used with a restore pulse optimized to minimize inflow artifacts^31^. In patients with sinus rhythm, the inversion pulse was performed every RR-interval, and for patients in AF during the scan the inversion pulse was performed every two RR-intervals. A Look-Locker was acquired prior to the LGE-Dixon scan to determine the optimal inversion time which ranged from 220 to 340 ms. No arrhythmia rejection algorithms were used. The inversion delay was determined using a Look-Locker scan to null healthy myocardium and the acquisition was triggered to ventricular systole defined by a time-resolved 4-chamber image.

### Image analysis

The quantified parameters were: LA volume (mL), LA EAT volume (mL), LA EAT area (cm^2^), LA fibrosis relative area (% of the LA wall surface), LA fibrosis absolute area (cm^2^), LA EAT- fibrosis relative overlap area (% of the LA wall surface), LA EAT—fibrosis absolute overlap area (cm^2^), left ventricular end diastolic volume (LVEDV) and ejection fraction (EF), the last two ones by using short-axis cine balanced steady-state free precession (bSSFP).

The water component of the mDixon images was used for LGE quantification while the fat component was used to quantify the EAT. Segmentations of the LA myocardium and EAT were performed by IS (3 years of cardiovascular imaging experience), using the Medical Imaging Interaction Toolkit (MITK, German Cancer Research Center, Heidelberg, Germany), while semi-automatic fibrosis, fat and overlap area quantification was implemented on a commercial workstation using MATLAB R2021b (The MathWorks, Natick, Massachusetts, USA). The 3D LA myocardium segmentation consisted of manual tracing of the left atrial wall (intramural) and exclusion of the left atrial appendage, left and right pulmonary veins (PV) antrums, and mitral valve. The LA volume was calculated by addition of LA area outlined in each transversal stack image of the LGE-Dixon sequence at ventricular systole. The semi-automatic fibrosis quantification was performed by obtaining a patient-specific threshold, based on the signal intensity (SI) of the mitral valve^15^. An example of the used method and a generated 3D left atrial map is presented in Fig. 3.

To calculate the spatial overlap between EAT and fibrosis, first a 3D mesh was generated from the LA wall segmentation. For each voxel of the LA wall a 3D vector orthogonal to the wall was then obtained based on the closest 3D mesh vertex normal. For a given voxel along the LA wall, if there was an EAT voxel along the orthogonal vector stretching 6 mm out from the LA, the LA wall voxel was considered adjacent to EAT. LA EAT area was calculated as LA wall voxels adjacent to EAT regardless of fibrosis classification, while the LA EAT-fibrosis overlap area included only the LA wall voxels which were defined as fibrosis based on the classification described in the previous paragraph, and adjacent to EAT (Fig. 3).

In order to facilitate comparison with studies from other research groups, we also performed fibrosis quantification based on image intensity ratio (IIR) with two different thresholds, 1.2 and 1.32^21,32^.

Heart failure is associated with the development and perpetuation of atrial fibrosis^33^, thus a regression analysis was performed regarding the fibrosis area, LA EAT-fibrosis overlap area, and LVEF.

### Statistical analysis

Continuous variables are expressed as mean ± standard deviation (SD). Non-normal distributed data are expressed as median (interquartile range). The group comparison for normally distributed data was performed by one-way ANOVA, and by Kruskal Wallis, Independent-samples Median Test and Wilcoxon signed rank test, for non-normally distributed data. A *P* value lower than 0.05 was considered significant. Ordinal logistic regression was used to analyse the variables’ capacity to predict the AF type. Logistic regression was used to analyse the possible relation between LA fibrosis area/LA EAT- fibrosis overlap area and LVEF. Statistical analysis was performed using IBM SPSS Statistics for Windows, version 29.0 (IBM Corp., Armonk, N.Y., USA).

## Data Availability

The data underlying this article cannot be shared publicly due to limitations in ethical permits. Anonymized data may be shared on reasonable request to the corresponding author.
